# Tailoring polyimide aerogel performance: the role of solid content in influencing microstructure, mechanical, thermal, and acoustic behaviors

**DOI:** 10.1039/d5ra06821e

**Published:** 2025-11-03

**Authors:** Wenge Wang, Shimin Wang, Dandan Li, Jinjun Yang, Hui Li

**Affiliations:** a Avic Composite Co., Ltd Beijing 101300 P. R. China; b State Key Laboratory of Advanced Fiber Materials, Center for Advanced Low-dimension Materials, College of Materials Science and Engineering, Donghua University Shanghai 201620 China lihui@dhu.edu.cn; c School of Materials Science and Engineering, Shanghai University of Engineering Science Shanghai 201620 PR China ldd@sues.edu.cn

## Abstract

In this study, polyimide aerogels (PIAs) with solid contents of 3%, 4%, 5%, and 6% were fabricated using non-directional freezing and freeze-drying techniques. A comprehensive investigation was carried out on their microstructures, mechanical, thermal insulation, and acoustic properties. As the solid content increased, the density and linear shrinkage of PIAs rose, while porosity decreased from 97.62% (PIA-3%) to 94.54% (PIA-6%). Microstructural analysis showed that high-solid-content PIAs had smaller pore sizes and denser network structures. Thermogravimetric analysis (TGA) tests indicated excellent thermal stability with initial decomposition temperatures above 500 °C, with little influence from solid content. In terms of mechanical properties, the Young's modulus and specific Young's modulus of high-solid-content aerogels were notably enhanced, with PIA-6% reaching 5703 kPa. The thermal conductivity of PIAs increased with solid content, from 40.81 mW m^−1^ K^−1^ for PIA-3% to 48.63 mW m^−1^ K^−1^ for PIA-6%, due to increased skeleton density and reduced porosity. Regarding acoustic properties, low-solid-content aerogels had better sound absorption, *e.g.*, a 2-cm PIA-3% sample had an average sound absorption coefficient of 0.602 and NRC of 0.393 in 250–6300 Hz, while for PIA-6%, they dropped to 0.416 and 0.311. Conversely, high-solid-content aerogels had superior sound insulation, with PIA-6% achieving an average STL of 30.38 dB compared to 11.94 dB for PIA-3%. Overall, the performance of PIAs can be tailored by adjusting the solid content, with low-solid-content materials suitable for sound absorption and high-solid-content ones for sound insulation and high-strength applications. This research offers new insights for developing high-performance acoustic materials and promotes the practical application of PIAs in aerospace, architectural acoustics, and traffic noise reduction.

## Abstract 2

In this study, polyimide aerogels (PIAs) with solid contents of 3%, 4%, 5%, and 6% were fabricated using non-directional freezing and freeze-drying techniques.

## Graphical abstract

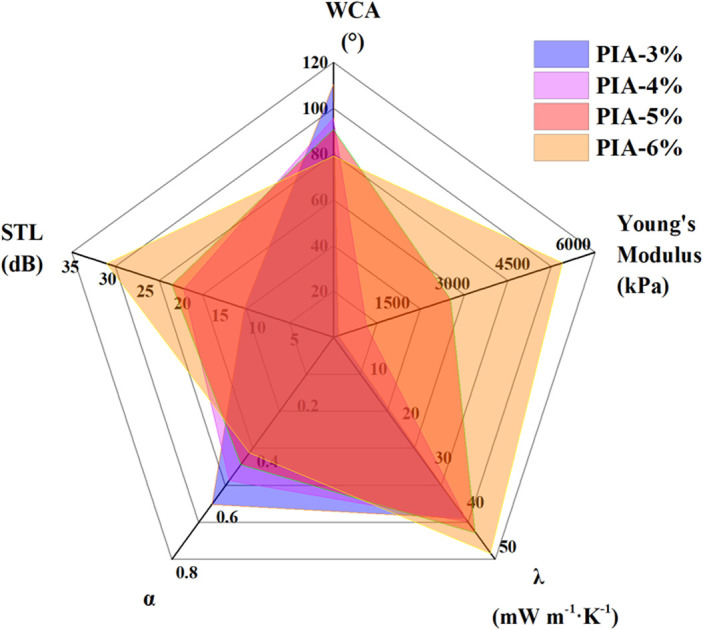

## 1. Introduction

Polyimide aerogels (PIAs), an emerging class of multifunctional micro–nano porous material, have garnered significant attention in advanced materials research due to its unique structure–property synergy.^1–3^ By integrating the intrinsic advantages of polyimides, exceptional tensile strength (>200 MPa) and thermal resilience (long-term stability >350 °C),^4–6^ with the hierarchical porosity of aerogels (ultralow density <0.08 g cm^−3^, thermal conductivity <50 mW m^−1^ K^−1^, and dielectric constant <1.8),^7–10^ PIAs achieve unprecedented performance benchmarks unattainable by conventional materials. In contrast to brittle inorganic SiO_2_ aerogels compromised by structural fragility,^11,12^ PIAs exhibit remarkable mechanical ductility,^13,14^ while surpassing organic counterparts such as polyurethane and cellulose aerogels in thermal stability.^15,16^ These exceptional attributes position PIAs as transformative candidates for aerospace acoustic and thermal shielding, flexible electronics encapsulation, and energy-efficient insulation systems, where the concurrent demand for mechanical robustness, thermal endurance, and ultralight architecture remains critical.^17–20^

Current fabrication strategies for PIAs predominantly encompass two methodologies: (a) sol–gel synthesis combined with supercritical CO_2_ drying, a process pioneered by NASA.^21–25^ This approach enables the formation of nanoscale network structures with controlled pore size distribution by adjusting precursor concentration and gel aging time. (b) Ice templating-assisted freeze-drying uses directional or non-directional freezing to guide the growth of ice crystals, forming anisotropic or non-anisotropic channels.^26,27^ This method is particularly suitable for fabricating functional aerogels with tailored gradient or hierarchical porosity. In the ice-templating approach, the solid content, typically defined as the mass fraction of the poly(amic acid) (PAA) or polyamide salt (PAAS) precursor in the solution, serves as a critical processing parameter. It directly governs the coupling relationship between mesostructural characteristics (porosity, specific surface area) and macroscopic properties (compressive strength, thermal conductivity) by modulating ice crystal growth dynamics and phase separation processes. Substantial evidence underscores the profound impact of solid content. For instance, Ma *et al.*^28^ demonstrated that when the solid content was increased from 4 wt% to 15 wt%, the compressive modulus increased by 4.7 times, while the porosity decreased by approximately 13%, showing a significant performance trade-off effect. In a similar vein, Hou *et al.*^29^ observed continuous improvements in the compressive strength and modulus of PI nanofiber aerogels as the solid content of nanofibers increased, with the 20 wt% sample achieving a tensile strength of 2.95 MPa. Furthermore, Wu *et al.*^30^ found that in silica aerogel powder (SAp)-reinforced PI composite aerogels, increasing the SAp content from 0% to 80% led to gradual decreases in density, shrinkage, and thermal conductivity.

While these studies confirm the pivotal role of solid content, a systematic understanding of its influence remains incomplete. Key challenges persist, including the unclear evolution mechanism of the multi-level internal microstructure across different solid contents and the lack of quantitative relationships linking solid content to the interplay of mechanical, thermal, and especially acoustic properties. Therefore, an in-depth investigation into the influence of solid content is crucial to reveal the underlying structure–property relationships, which will provide a solid theoretical basis and practical guidance for optimizing the preparation process and developing PIAs with superior comprehensive performance.

In this study, the PIAs system constructed with 4,4′-diaminodiphenyl ether (ODA) and 3,3′,4′-biphenyltetraformic anhydride (BPDA) was taken as the research object. The ice template assisted freeze-drying technology was adopted to systematically investigate the influence mechanism of solid content (3–6 wt%) on the formation of multi-level pore structure of aerogel and its influence on performance. This work elucidated the correlations among solid content, pore structure, and performance, providing theoretical guidance for developing porous materials with integrated noise reduction and thermal insulation functions.

## 2. Experimental section

### 2.1 Materials

*N*-Methylpyrrolidone (NMP, AR), 4,4′-diaminodiphenyl ether (ODA, 98%), 3,3′,4,4′-biphenyl tetracarboxylic acid dianhydride (BPDA, 98%), triethylamine (TEA, AR) and ethanol (EtOH, AR) were purchased from Shanghai Taitan Technology Co., Ltd. BPDA and ODA should be treated in a vacuum oven at 80 °C for 24 h before use. The deionized water was homemade by the laboratory and used throughout the experiments.

### 2.2 Synthesis of PAAS

**Fig. 1:**
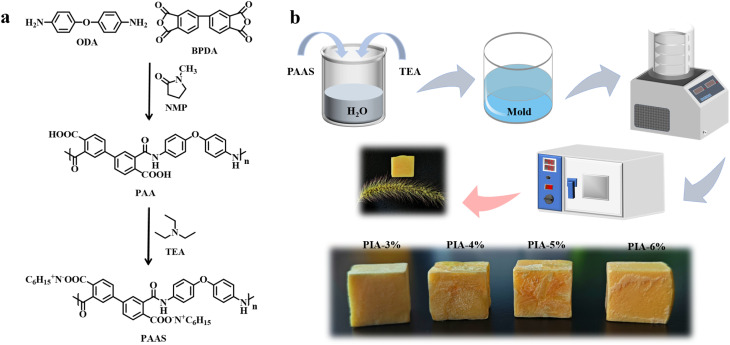
Chemical synthesis of PAAS (a); fabrication of PIAs (b).

The PAAS was synthesized through a controlled stepwise polymerization process under inert atmosphere. Specifically, ODA was first dissolved in anhydrous NMP within a nitrogen-purged three-neck flask maintained at 25 ± 2 °C. Equimolar BPDA was then added incrementally over 15 min under mechanical stirring, followed by continuous reaction for 8 h to obtain a viscous PAA solution with 13 wt% solid content. Subsequent neutralization was performed by dropwise addition of TEA with simultaneous adjustment of stirring speed to maintain homogeneous mixing while preventing localized viscosity spikes. This critical phase spanned 3 h until complete PAAS formation. The resultant PAAS solution was precipitated into a 10-fold volume excess of ethanol to induce phase separation, yielding a fibrous white precipitate. The crude product underwent mechanical pulverization, followed by three wash cycles with fresh ethanol to remove residual NMP and oligomers, and finally dried under vacuum at 60 °C for 24 h to obtain the PAAS powder. A schematic flowchart of this chemical synthesis is illustrated in Fig. 1a.

### 2.3 Fabrication of PIAs with controlled solid contents

The PIAs were fabricated through a four-stage process: solution formulation, isotropic freezing, freeze-drying, and thermal imidization. First, PAAS aqueous solutions with precisely controlled solid contents (3, 4, 5, and 6 wt%) were prepared by mixing stoichiometric quantities of PAAS powder and deionized water in a reaction vessel maintained at 25 ± 0.5 °C. TEA was then gradually added to the mixture under stirring until complete dissolution of the solids was achieved. The homogeneous PAAS solutions were poured into designated molds and subjected to isotropic freezing at −18 °C for 24 h. Subsequently, the frozen samples were lyophilized in a freeze-dryer under a vacuum of 1 Pa for 72 h to obtain porous PAAS frameworks. Finally, thermal imidization was carried out in a stepwise heating program: 100 °C for 1 h, 150 °C for 1 h, 200 °C for 2 h, and 230 °C for 2 h, yielding PIAs. The resulting PIAs were labeled as PIA-3%, PIA-4%, and PIA-5% based on their initial solid content.

### 2.4 Characterization

The chemical composition of PIAs samples was analyzed using a Fourier transform infrared (FT-IR, Bruker, GER) spectrometer in the wavelength range 400–4000 cm^−1^ with 32 scans at 4 cm^−1^ resolution. The internal microstructure of the samples was observed *via* field-emission scanning electron microscope (FE-SEM, Regulus 8230, Hitachi, JP) after gold spraying. PIAs density (*ρ*) was calculated according to eqn (1)1
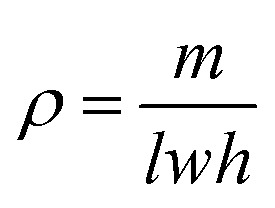
where geometric dimensions (length *l*, width *w*, height *h*) were measured using a digital micrometer, and mass (*m*) was determined *via* analytical balance.

Porosity was calculated according to eqn (2):2
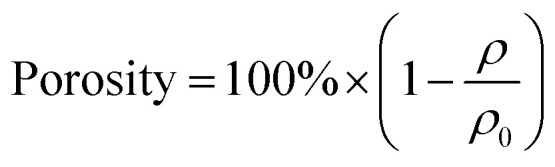
where *ρ* is obtained from eqn (1) and *ρ*_0_ = 1.38 g cm^−3^ represents the bulk density of fully dense polyimide.

Dimensional shrinkage was calculated according to eqn (3):3
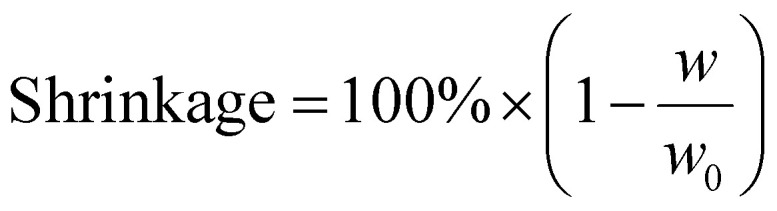
where *w* is the width of the aerogel which was measured at room temperature after freeze-drying (1 Pa, 72 h) and thermal imidization up to 230 °C for 2 h, and *w*_0_ is the initial mold diameter.

Thermal properties of PIAs were evaluated by thermogravimetric analysis (TGA, Discovery 550, TA Instruments, USA) under nitrogen atmosphere at a heating rate of 10 °C min^−1^ from 30–800 °C. Surface hydrophobicity was assessed *via* static water contact angle measurements (OCA40Micro, DataPhysics, CHN) using 5 μL droplets. Uniaxial compression tests of PIAs samples were performed on cylindrical specimens by a universal testing machine (Instron 5966, USA) at 2 mm min^−1^ strain rate. Compression Young's modulus (*E*) was calculated as the average of five values. Specific Young's modulus, as a key indicator of structural efficiency, was calculated as the ratio of the compressive Young's modulus to the density, representing the stiffness per unit mass. Thermal conductivity (*λ*) of the samples was measured *via* transient plane source method (Hot Disk TPS 2500 S, SE) at 25 °C, where required two identical surfaces of samples to ensure smooth contact with the probe. Acoustic performance was characterized using impedance tubes (SW477/SW422, BSWA Technology, CHN). Samples were machined to 29.9 mm (high-frequency: 1600–6300 Hz) and 99.6 mm (low-frequency: 250–1600 Hz) diameters, ensuring airtight contact with tube walls. Sound absorption coefficients (*α*) were averaged over five measurements per frequency band.

## 3. Results and discussion

### 3.1 Chemical and physical properties of PIAs with controlled solid contents

**Fig. 2:**
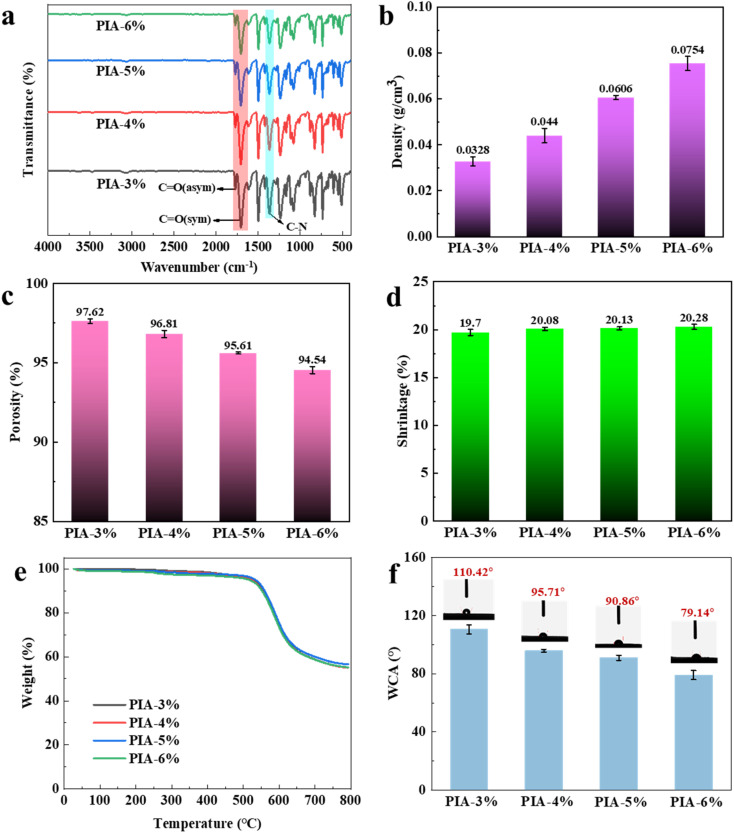
FTIR curves (a), density (b), porosity (c), shrinkage (d), TGA (e) and water contact angle (f) of PIAs.

Fig. 2a presents the FT-IR spectra of PIAs synthesized with systematically varied solid contents (3–6 wt%), where characteristic peaks correspond to distinct chemical bonds. All spectra exhibit three diagnostic absorption bands corresponding to imide ring vibrations.^31^ Specifically, the peak at 1775 cm^−1^ corresponds to the asymmetric stretching vibration of C=O in the imide ring, while the peak at 1710 cm^−1^ arises from the symmetric stretching of C=O. The band at 1371 cm^−1^ is assigned to the C–N bond within the imide ring. Additionally, the positional consistency of these characteristic peaks across samples confirms successful imidization and preservation of polyimide chemical architecture regardless of solid content variations.

The physical properties of PIAs revealing the solid content-dependent evolution, including density, porosity, and linear shrinkage, were characterized, as shown in Fig. 2b–d. Due to the low solid content in the precursor solution, PIA-3% sample (3 wt% solid content) achieves an ultralow density of 0.0328 g cm^−3^ and ultrahigh porosity of 97.62%, accompanied by a significant linear shrinkage of 19.70% after freeze-drying and thermal imidization. When the solid content increases from 3 wt% to 6 wt%, PIA gradually densifies, the increase in shrinkage rate gradually increases, and the porosity gradually decreases. The shrinkage primarily occurs during two stages: freeze-drying and thermal imidization. (a) During freeze-drying, the sublimation of ice crystals induces inward collapse of the sample under vacuum. For PIAs with lower PAAS solid content (*e.g.*, PIA-3%), this collapse is partially reversible. However, higher PAAS solid content leads to irreversible structural collapse, resulting in increased shrinkage. (b) In the thermal imidization stage, dehydration and imide ring formation further drive shrinkage. Higher PAAS solid content enhances the degree of imidization, thereby amplifying shrinkage. For instance, the PIA-6% sample (6% solid content) displays a higher density of 0.0754 g cm^−3^ and a slightly elevated linear shrinkage of 20.28%, while its porosity decreases to 94.54%. The reduced porosity at higher solid contents is attributed to the diminished proportion of ice-templated pores, as the higher solid-to-solvent ratio limits pore formation during drying.^32^

TGA curves under nitrogen atmosphere were displayed in Fig. 2e, showcasing thermal stability characteristics of PIAs. All samples demonstrate outstanding thermal resistance, with the temperatures corresponding to 5% weight loss (*T*_d5%_) measured at 525 °C for PIA-3%, 523 °C for PIA-4%, 533 °C for PIA-5%, and 515 °C for PIA-6%, respectively. Notably, the TGA profiles across different solid contents display striking consistency, indicating that the thermal stability of these aerogels is primarily governed by the intrinsic chemical structure of polyimide rather than macroscopic morphological features like porosity or density. The rigid aromatic backbones and thermally stable imide rings in the molecular chains dictate the decomposition behavior, which relies on the bond dissociation energies of C–N, C=O, and aromatic C–C bonds. Despite observable variations in macroscopic properties such as density and pore structure among samples with different solid contents, the uniformity of chemical bonding at the molecular level ensures remarkably similar thermal degradation trends. This highlights the dominant role of intrinsic chemical structure over physical morphology in determining the thermal stability of these polyimide-based aerogels.

Fig. 2f illustrates the water contact angles (WCAs) of PIAs with varying solid contents, revealing a notable decrease in hydrophobicity as solid content increases. The WCA decreases systematically from 110.42° for PIA-3% to 79.14° for PIA-6%, a trend attributed to the denser microstructural network formed at higher solid loadings. This compact architecture induces polar functional groups, such as the carbonyl groups (C=O) in imide rings, to orient toward the aerogel surface, thereby increasing surface energy and promoting hydrophilic interactions with water. Simultaneously, the reduced porosity and smaller pore diameters associated with higher solid contents enhance capillary action, facilitating liquid penetration into the network. These combined effects, surface chemical reorientation and intensified capillary forces, drive a progressive reduction in surface hydrophobicity, manifested as lower contact angles across the series. This demonstrates a clear correlation between solid content-regulated microstructural features and surface wettability in PIAs.

### 3.2 Microstructure analysis of PIAs with controlled solid contents

**Fig. 3:**
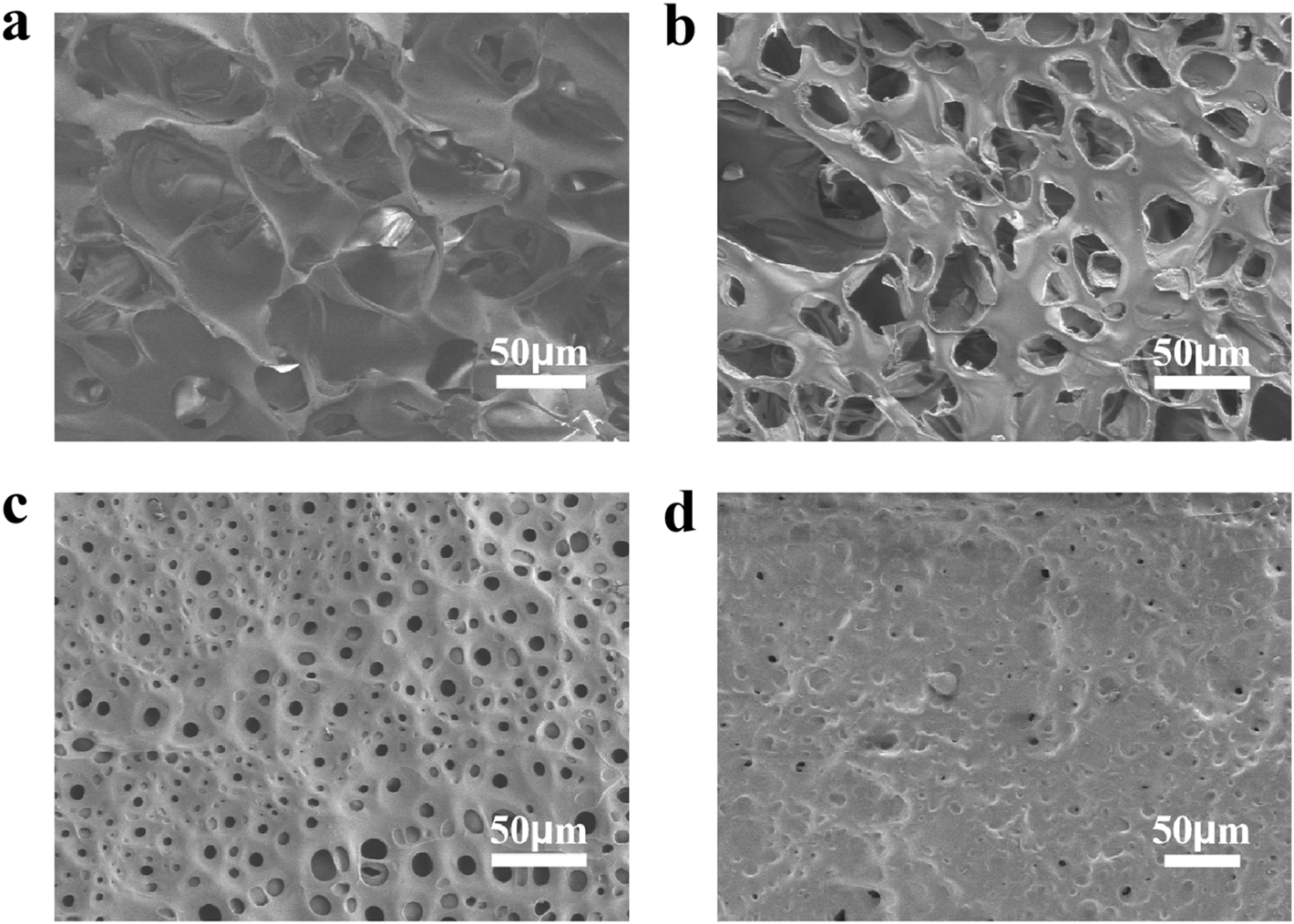
SEM images for PIA-3% (a), PIA-4% (b), PIA-5% (c) and PIA-6% (d).

Fig. 3 presents the SEM micrographs of PIAs fabricated *via* non-directional freezing, revealing a distinct microstructural architecture. All PIAs samples exhibit an irregular porous network structure that stands in stark contrast to the unidirectionally aligned pore structures typically produced by liquid nitrogen-assisted directional freezing.^33^ This random porous network gives rise to tortuous transport pathways that prolong or impede the transmission of sound waves and thermal energy, which is the key attributes for multifunctional insulation and noise-reduction applications. Notably, the PIAs feature smooth inner surfaces of the pore walls, a characteristic that may influence surface-related properties such as acoustic wave transmission and heat transfer dynamics. A systematic decrease in pore size is observed with increasing solid content, a phenomenon directly linked to the freezing behavior of the precursor solutions. A systematic decrease in pore size is observed with increasing solid content, a phenomenon directly linked to the freezing behavior of the precursor solutions. At lower solid contents (*e.g.*, 3 wt%), a higher solvent-to-polymer ratio allows for unimpeded growth of ice crystals during the freezing process, resulting in larger ice templates and correspondingly larger pores after solvent sublimation. Conversely, elevated solid contents (*e.g.*, 6 wt%) lead to a denser polymer matrix that physically hinders ice crystal expansion; the reduced solvent availability and increased macromolecular entanglement restrict ice growth, yielding smaller ice crystals and, consequently, narrower pore dimensions in the final aerogel structure. This solid content-dependent pore size regulation highlights the critical role of precursor solution composition in tailoring the microstructural features of non-directionally frozen PIAs.

### 3.3 Mechanical properties of PIAs with controlled solid contents

**Fig. 4:**
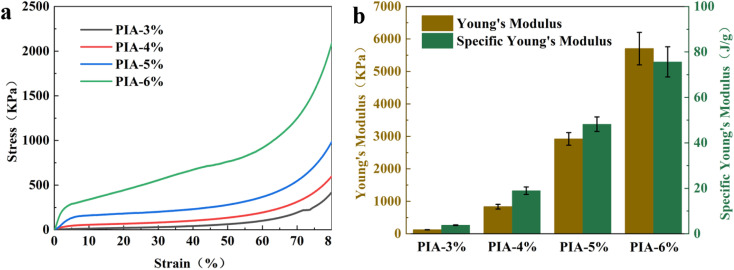
Compression stress–strain curves (a); Young's modulus and specific Young's modulus of PIAs (b).

The compressive stress–strain (*σ*–*ε*) curves of PIAs with varying solid contents are presented Fig. 4a. The results reveal a marked upward trend in the *σ*–*ε* curves as the solid content increases. All aerogel samples exhibit three distinct compression stages: (a) a linear elastic deformation region (0 < *ε* < 5%, except PIA-3%), (b) a relatively flat plateau region (5% < *ε* < 60%), and (c) a steep densification stage (*ε* > 60%) characterized by a rapid stress surge. As illustrated in Fig. 4b, both the Young's modulus and specific Young's modulus of the PIAs increase progressively with higher solid content. Notably, the PI-6% aerogel demonstrates the highest Young's modulus (5703 kPa) and specific Young's modulus (76 J g^−1^) among all samples. This enhancement is attributed to the increased skeletal density of the PI-6% aerogel, which strengthens intermolecular interactions and promotes a more tightly crosslinked three-dimensional network.^34,35^ Concurrently, the reduced porosity and smaller pore size at elevated solid content minimize internal structural defects, thereby enhancing rigidity and contributing to the superior compressive modulus.

### 3.4 Thermal insulation of PIAs with controlled solid contents

**Fig. 5:**
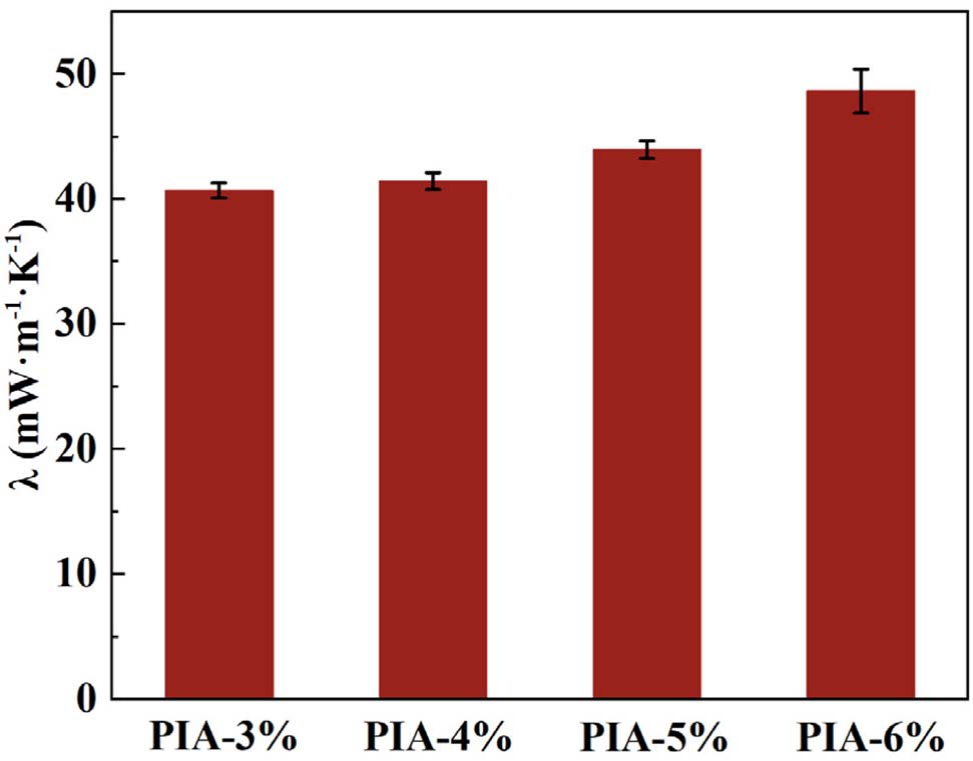
Thermal conductivity of PIAs.

The excellent thermal insulation properties of PIAs prepared *via* non-directional freeze-drying are demonstrated in Fig. 5. The thermal conductivity of PIAs with varying solid contents ranges from 40.81 to 48.63 mW m^−1^ K^−1^. At 25 °C, the PIA-3% sample exhibits an ultralow thermal conductivity of 40.81 mW m^−1^ K^−1^, which increases to 48.63 mW m^−1^ K^−1^ for the PIA-6% sample as the solid content rises. This positive correlation between solid content and thermal conductivity is attributed to microstructural densification and its direct impact on heat transfer mechanisms. The increase in thermal conductivity is primarily driven by the enhancement of solid-phase conduction. Higher solid content leads to microstructural densification, characterized by increased skeleton density, thicker pore walls, and a more continuous three-dimensional network. This improved connectivity suppresses phonon scattering at interfaces and establishes more efficient pathways for heat transfer through the polymer matrix.^36^ Furthermore, the reduction in porosity diminishes the volume fraction of trapped air, which possesses a low thermal conductivity of ∼26 mW m^−1^ K^−1^, thereby increasing the relative contribution of the higher-conductivity PI phase. Concurrently, the contribution of thermal radiation is influenced in a competing manner. While radiation typically becomes significant at higher temperatures, the increased solid content and associated structural complexity enhance the infrared extinction coefficient of the aerogel. The higher density of pore walls and solid–gas interfaces provides more scattering centers, which likely suppresses radiative heat transfer to some extent. Critically, the contribution of gas-phase convection to the overall heat transfer is considered negligible. The pore sizes in PIAs, typically in the sub-micron to several micron range, result in a pore-size-dependent Rayleigh number far below the critical value required to initiate convective flows. Therefore, the gas phase remains essentially stagnant across all samples. In summary, the observed rise in thermal conductivity with increasing solid content results from the dominance of enhanced solid conduction and the increased relative contribution of gas conduction, which collectively outweigh the minor suppressing effect on radiation and the consistently negligible convection.

### 3.5 Acoustic performances of PIAs with controlled solid contents

**Fig. 6:**
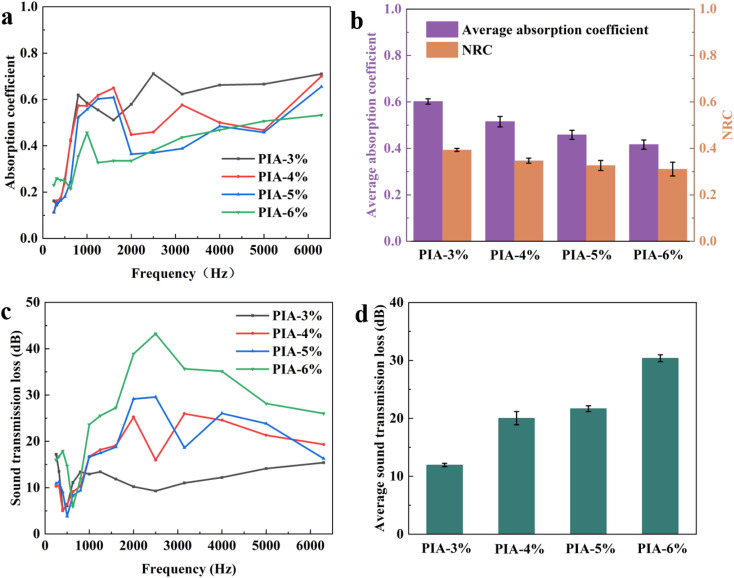
The relationship between absorption coefficient and frequency (a), average absorption coefficient of 250–6300 Hz and NRC (b), the relationship between sound transmission loss and frequency (c) and sound transmission loss of 250–6300 Hz (d) of PIAs.

Thanks to their customizable porous structure, PIAs are widely used in the field of sound absorption and noise reduction.^37,38^ To further investigate the acoustic performance, 2-cm-thick PIAs were prepared, and their sound absorption coefficient and sound transmission loss (STL) were analyzed. For sound absorption properties, the study reveals a declining trend in sound absorption coefficient curves with increasing solid content. The average sound absorption coefficient (calculated over 250–6300 Hz) and noise reduction coefficient (NRC, determined as the average of 1/3-octave bands at 250 Hz, 500 Hz, 1000 Hz, and 2000 Hz) are summarized in Fig. 6a and b. The PIA-3% sample exhibits a high average sound absorption coefficient of 0.602 and an NRC of 0.393, whereas these values decrease to 0.416 and 0.311, respectively, for the PIA-6% sample. Both parameters diminish progressively with higher solid content. This trend is attributed to alterations in the porous structure and acoustic impedance matching at elevated solid contents. Firstly, the sound absorption mechanism relies on pore-mediated energy dissipation, where sound waves penetrate hierarchical pores and are attenuated *via* viscous air friction and pore-wall vibrations. Higher solid content reduces porosity, narrows pore size distribution, and weakens pore connectivity, thereby limiting sound wave penetration depth and scattering pathways, which compromises energy dissipation efficiency. Secondly, increased solid content enhances skeleton density and elastic modulus, resulting in greater material rigidity. This suppresses damping effects from skeleton vibrations induced by sound waves, reducing the conversion of acoustic energy to mechanical energy. Additionally, acoustic impedance matching plays a critical role: low-solid-content aerogels exhibit impedance closer to air, facilitating sound wave entry, whereas high-solid-content materials display significantly higher impedance, causing more interfacial reflection rather than internal wave propagation.^39^

For sound insulation performance, in contrast to the sound absorption coefficient curves, the STL curves of PIAs show an upward trend with increasing solid content. The average STL values over 250–6300 Hz are summarized in Fig. 6c and d. The PIA-3% sample exhibits an average STL of only 11.94 dB at 2 cm thickness, while the PIA-6% sample achieves a significantly higher average STL of 30.38 dB. This improvement stems from structural densification, acoustic impedance shifts, and altered sound wave propagation paths at higher solid contents. Firstly, increased solid content directly enhances skeleton density and mechanical strength, reduces internal porosity, and thickens pore walls, creating greater rigid resistance to sound wave penetration. The elevated elastic modulus of high-solid-content aerogels enhances sound wave reflection, particularly for mid-to-high-frequency waves (1000–5000 Hz). Secondly, STL is closely linked to acoustic impedance. High-solid-content aerogels exhibit impedance closer to solid materials, exacerbating impedance mismatch with air and promoting interfacial reflection rather than transmission. Furthermore, reduced pore connectivity at higher solid contents leads to more closed or semi-closed pores, which amplify sound wave scattering and multiple reflections, prolonging propagation paths and dissipating acoustic energy. For a more intuitive comparison, the average sound absorption coefficient, NRC, and STL of the different aerogels are summarized in Table 1.

**Table 1:** The average absorption coefficient, NRC and average STL of different aerogels

Samples	Thickness (cm)	Frequency (Hz)	*α*	NRC	STL (dB)
PIA-3% (this work)	2.0	250–6300	0.602	0.393	11.94
PIA-6% (this work)	2.0	250–6300	0.416	0.311	30.38
PI/SiO_2_-6% (ref. 33)	1.8	500–6300	—	—	32.98
K1.0S1.0G1.0WS1.0 (ref. 40)	—	125–4000	0.335	—	—
MPL-ANFs^41^	2.0	50–6300	0.586	0.52	—
CAs^42^	1.0	50–5000	0.337	0.32	—
RGO@ANF-40 (ref. 43)	1.0	—	—	0.20	—

### 3.6 Comprehensive performance comparison of PIAs

**Fig. 7:**
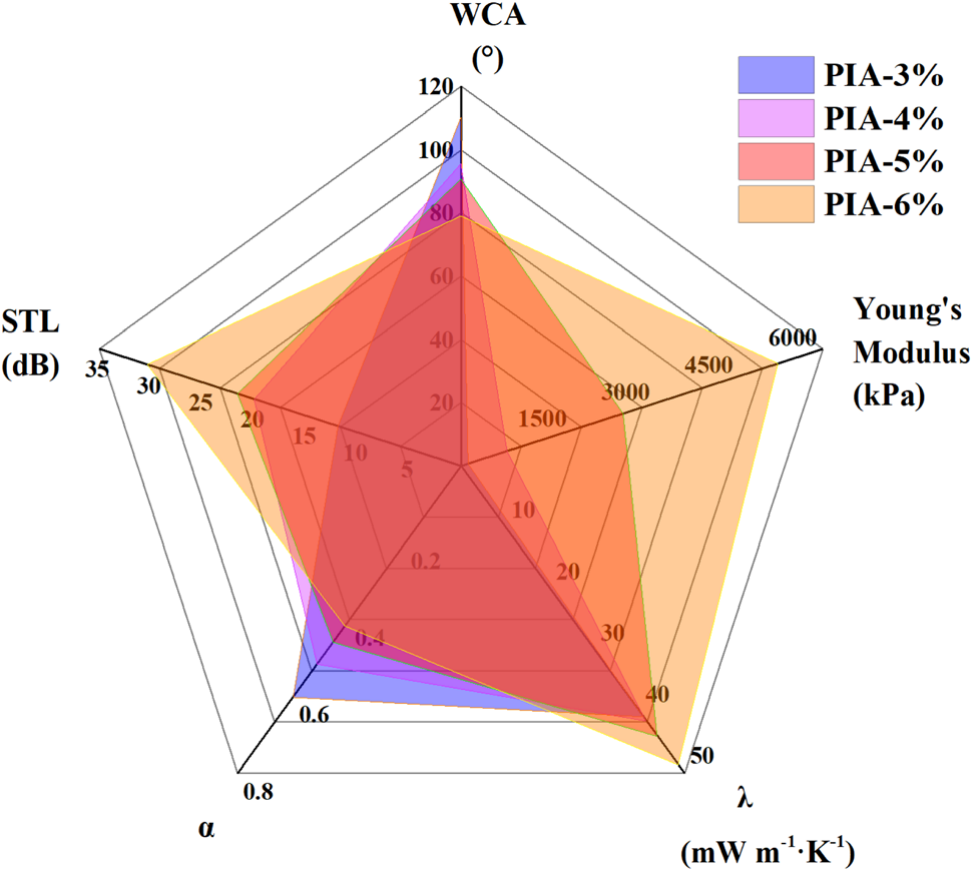
Comprehensive performance comparison of PIAs with different solids content.

The comprehensive performances of PIAs dependent on the change of solid content are compared, and the results are presented in Table 2 and Fig. 7. The solid content in the synthesis of PIAs exerts a profound influence on their multifunctional performance, exhibiting a synergistic interplay between structural and surface effects. As solid content increases, the WCA of the aerogels decreases in a systematic manner, reflecting a transition from hydrophobic to increasingly hydrophilic surface characteristics. Concurrently, mechanical strength improves progressively, attributed to the formation of a denser polymer network and reduced pore connectivity induced by higher solid loadings. In terms of thermal insulation, aerogels synthesized with higher solid contents exhibit increased thermal conductivity, primarily due to microstructural densification that enhances solid-phase heat transfer pathways. The acoustic performance also indicates a clear trade-off relationship: the higher the solid content, the lower the sound absorption coefficient will be, while the sound insulation performance will improve accordingly. This versatility enables the selection of materials for targeted applications, from scenarios demanding sound absorption to those requiring sound reflection. The denser microstructures at higher solid contents enhance resistance to sound wave propagation, leveraging reduced pore interconnectivity and increased structural rigidity to impede acoustic transmission. These observations underscore the trade-offs and interdependencies among surface wettability, mechanical integrity, and acoustic behavior governed by solid content, offering critical insights for tailoring polyimide aerogels to meet specific application demands. By optimizing solid content, researchers can systematically balance these properties to develop aerogels with customized performance profiles for diverse engineering and technological contexts.

**Table 2:** Comprehensive performances of PIAs with different solids content

Samples	Density (g cm^−3^)	Porosity (%)	Shrinkage (%)	WCA (°)	Young's modulus (kPa)	Specific Young's modulus (J g^−1^)	*T*_d5%_ (°C)	*λ* (mW m^−1^ K^−1^)	*α*	STL (dB)
PIA-3%	0.0328^a^	97.62^a^	19.70^a^	110.42^a^	125^a^	3.8^a^	525^a^	40.81^a^	0.602^a^	11.94^a^
PIA-4%	0.0440	96.81	20.08	95.71	834	19.0	523	41.45	0.515	20.03
PIA-5%	0.0606	95.61	20.13	90.86	2921	48.2	533	43.96	0.458	21.67
PIA-6%	0.0754	94.54	20.28	79.14	5703	76.0	515	48.63	0.416	30.38

a Relevant data previously reported by the laboratory in the literature.^37^

## 4. Conclusion

In summary, a series of PIAs with different solid contents (3%, 4%, 5%, 6%) were prepared through non-directional freezing and freeze-drying techniques, and their microstructures, mechanical properties, thermal insulation, and acoustic characteristics were systematically studied. The research results show that as the solid content increases, the density and linear shrinkage rate of the PIAs gradually increase, while the porosity decreases from 97.62% (PIA-3%) to 94.54% (PIA-6%). Microstructural analysis also reveals that the PIAs with high solid content have smaller pore sizes and a denser network structure. Meanwhile, through TGA testing, it is found that the PIAs have excellent thermal stability, with the initial decomposition temperature all higher than 500 °C, and the solid content has little effect on the thermal stability. From the mechanical property tests of the obtained PIAs, it is found that the Young's modulus and specific Young's modulus of the aerogels with high solid content are significantly improved, and the Young's modulus of PIA-6% reaches 5703 kPa. In terms of thermal insulation performance, the thermal conductivity of the PIAs increases with the increase of the solid content. The thermal conductivity of the PIA-3% sample is 40.81 mW m^−1^ K^−1^, while that of the PIA-6% sample increases to 48.63 mW m^−1^ K^−1^, which is attributed to the enhanced thermal conduction ability caused by the increase in the density of the skeleton and the decrease in the porosity. In terms of acoustic performance, the aerogels with low solid content have better sound absorption performance. The average sound absorption coefficient of the 2 cm PIA-3% sample in the frequency range of 250–6300 Hz is 0.602, and NRC is 0.393, while the average sound absorption coefficient and NRC of the 2 cm PIA-6% sample decrease to 0.416 and 0.311 respectively. On the contrary, the aerogels with high solid content have better sound insulation performance. The average STL of PIA-6% reaches 30.38 dB, which is significantly higher than 11.94 dB of PIA-3%. In conclusion, the performance of the PIAs can be regulated by the solid content to meet different application scenarios. The materials with low solid content are more suitable for sound absorption applications, while the materials with high solid content are more suitable for scenarios with high requirements for sound insulation and mechanical strength. This study provides new ideas for the development of high-performance acoustic materials and promotes the practical application of PIAs in fields such as aerospace, architectural acoustics, and traffic noise reduction.

## Author contributions

Wenge Wang: data curation, formal analysis, writing – original draft. Shimin Wang: data curation, formal analysis. Dandan Li: visualization, conceptualization, writing – review & editing. Jinjun Yang: conceptualization, formal analysis, visualization. Hui Li: methodology, resources, visualization.

## Conflicts of interest

The authors declare that they have no known competing financial interests or personal relationships that could have appeared to influence the work reported in this paper.

## Data Availability

Data will be made available on request.
